# A new c.681dup *RUNX1* variant in familial leukemia

**DOI:** 10.1007/s10689-026-00550-7

**Published:** 2026-04-06

**Authors:** Maria Crocioni, Carlotta Nardelli, Anair Graciela Lema Fernandez, Valentina Bardelli, Valentina Pierini, Caterina Matteucci, Eloise Beggiato, Matteo Olivi, Valentina Vigliani, Alessandra Pelle, Giuseppe Lanzarone, Cristina Mecucci

**Affiliations:** 1 Institute of Hematology and Center for Hemato-Oncology Research, University of Perugia and Santa Maria della Misericordia Hospital, Perugia, Italy; 2 Division of Hematology, Department of Molecular Biotechnology and Health Sciences, University of Turin, Turin, Italy; 3 SSD Ematologia e Malattie Trombotiche, ASL Città di Torino, Turin, Italy; 4 Dipartimento di scienze mediche, Università degli studi di Torino, SC Genetica Medica U, AOU Città della salute e della scienza di Torino, Turin, Italy; 5 SC Genetica Medica U, AOU Città della salute e della scienza di Torino, Molinette Hospital, Turin, Italy

**Keywords:** Leukemia predisposition, *RUNX1* variant, Friedreich Ataxia

## Abstract

Constitutional *RUNX1* gene variants are associated with Familial Platelet Disorder (FPD) and predispose to a variety of hematological malignancies, included Acute Myeloid Leukemia (AML) and, albeit less frequently, Acute Lymphoblastic Leukemia (ALL). In this study, we report on a proband with primary diagnosis of AML, followed by T-ALL after transplant, and a positive familial history for leukemia over three generations. A new heterozygous germline pathogenic *RUNX1* (c.681dup, p.(Leu228ThrfsTer33)) variant was found in the proband and his affected mother.

**Supplementary Information:**

The online version contains supplementary material available at 10.1007/s10689-026-00550-7.

## Introduction

RUNX1, encoded by the *RUNX1* gene at 21q22.12 is the human homolog of the Drosophila *runt*, sharing the runt-homology domain (RHD) with both *RUNX2* and *RUNX3* related genes. RUNX1 is constitutively expressed in the hematopoietic cells [1], and it is frequently mutated in leukemia [2]. It is responsible for the DNA binding by the RUNX1-CBFβ complex [2] which regulates differentiation steps of hematopoiesis and stem cell generation during development [3]. Constitutional *RUNX1* mutations are associated with a Familial Platelet Disorder (FPD) predisposing to a variety of hematological malignancies, mostly Myelodysplastic Syndromes (MDS) and Acute Myeloid Leukemia (AML) [4, 5], and, less frequently, T- and B- Acute Lymphoblastic Leukemia (ALL) [6, 7] and Lymphomas [8]. Inherited heterozygous *RUNX1* variants, however, are not sufficient for the development of the leukemic phenotype. Additional somatic hits, such as mutation or deletion of the *RUNX1 wild-type* allele, pathogenic variants affecting other genes, or acquired chromosome rearrangements, have been identified at time of leukemia development [9, 10]. Here, we describe a novel pathogenic *RUNX1* variant in a Family with autosomal dominant leukemia and coexistence of the autosomal recessive Friedreich Ataxia (FRDA).

## Proband

A 43 years-old male was admitted at the Città della Salute e della Scienza in Turin in November 2022 because of leucocytosis and thrombocytopenia. Laboratory findings were as follows: Hb 11.2 gr/dl, WBC 36.160 × 10^9^/l with 79% blasts, PLTs 11.00 × 10^9^/l, AST 414 UI/l (ULN 50), ALT 277 UI/l (ULN 50), LDH 3185 U/l (ULN 280). Bone Marrow (BM) aspirate showed 74% of blasts. Immunophenotype identified a predominant cell population positive for myeloid and immature antigens (CD13, CD33, weak MPO, CD34, weak CD38, HLA-DR, weak CD45), and also 8% of hypogranulated blasts, expressing CD7 and cyCD79a but negative for MPO, cyCD3, CD10, CD1a, CD2, CD5, CD3 and CD19. The BM biopsy showed 90% of cellularity and diffuse infiltration from hypogranulated blasts and expression of CD33, CD34, CD7, CD79a and MPO, supporting the diagnosis of AML without maturation. Additionally, histological analysis on a voluminous anterior mediastinal mass, documented at CT scan, showed extramedullary disease localization. The final diagnosis was AML. Chemotherapy was administered using a FLAI-Venetoclax (V-FLAI) scheme.

**Fig. 1:**
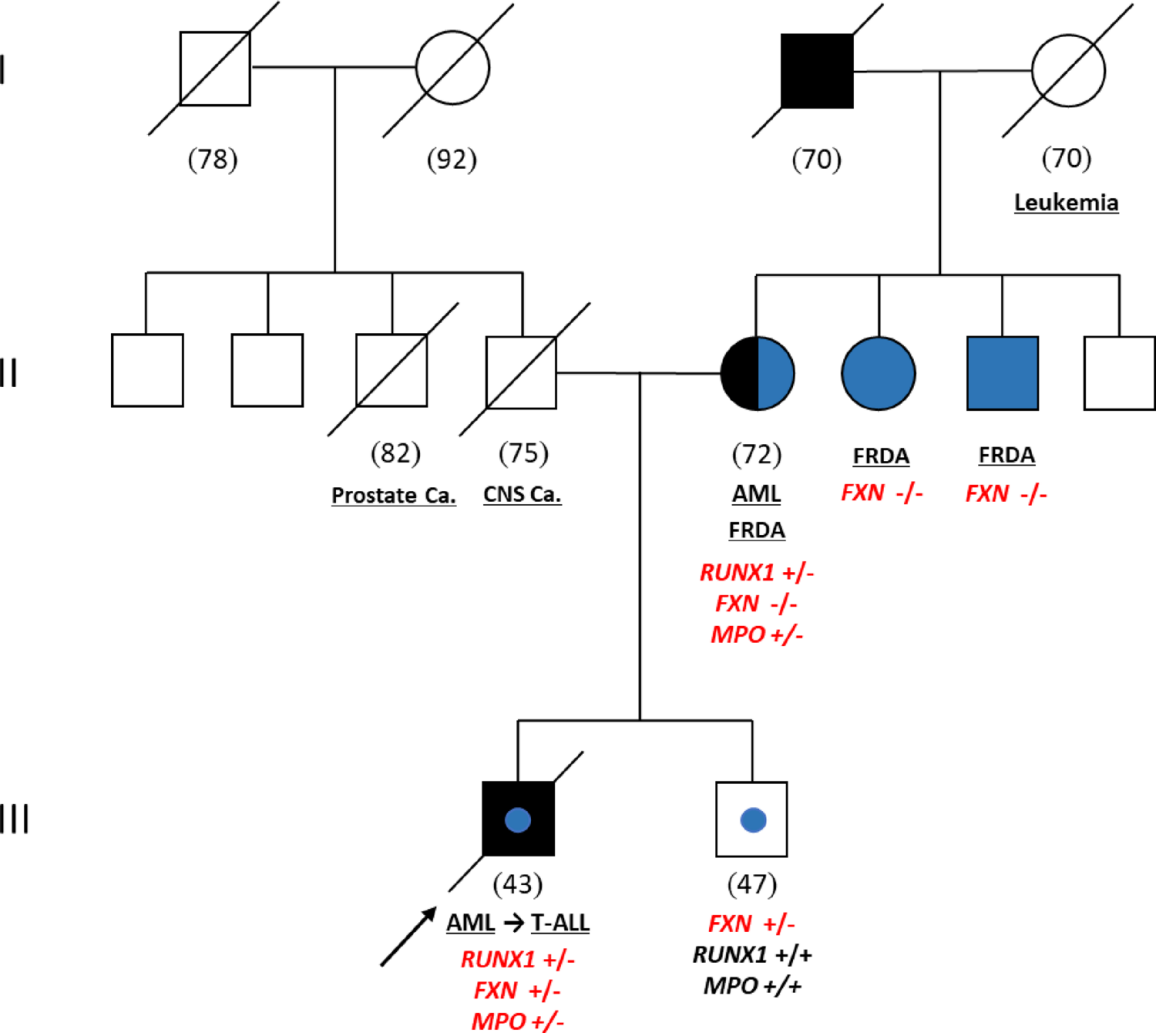
Family pedigree. Proband is indicated by the arrow. Mutational status is shown as + (wild-type allele) and − (mutant allele) for *FXN* trinucleotide repeat expansion, *RUNX1* c.681dupA and *MPO* c.2031–2 A > C. Black: relatives with positive history for hematological malignancies. Blue: relatives with FRDA (blue circlet for the carriers). White: no information or healthy individuals. The numbers in brackets in Members investigated represent the age at diagnosis for the proband and his mother and the age at time of sampling for the healthy brother. All the other family members are reported with the age of death. FRDA, Friedreich’s Ataxia; AML. Acute Myeloid Leukemia; T-ALL, T-Cells Acute Lymphoblastic Leukemia; Prostate ca., Prostate cancer; CNS ca.; Central Nervous System cancer.

**Fig. 2:**
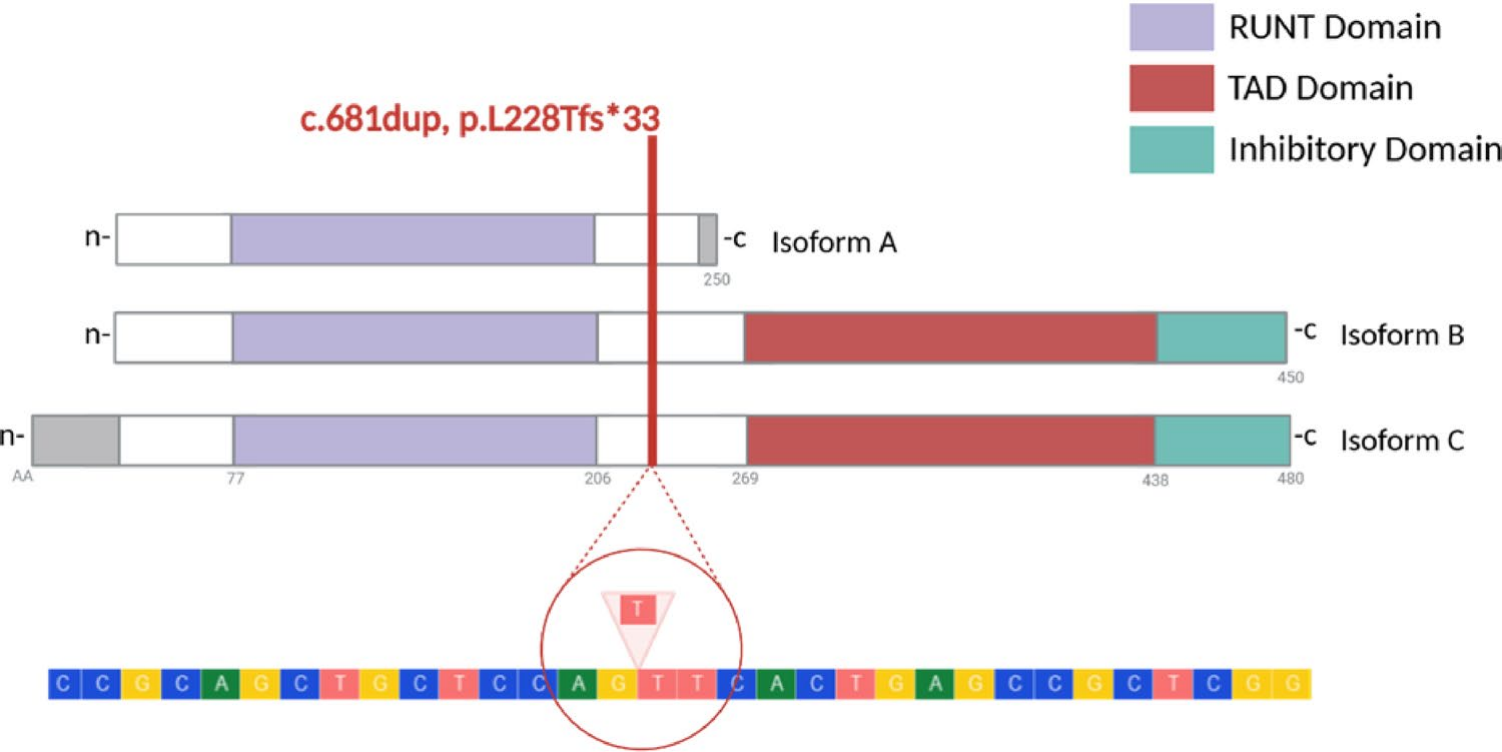
Schematic representation of the *RUNX1 *c.681dup variant localization at the different RUNX1 isoforms and site of thymine duplication within the genomic sequence. Created in BioRender. Lema, A. (2025) https://BioRender.com/xfuy56w

The family history disclosed hematological malignancies in close relatives (Fig. 1). In particular, the proband’s mother, aged 72, was under treatment in another Centre because of refractory AML, and the grandfather from the maternal side had died at the age of 70 because of undefined leukemia. Germline and somatic analysis in both the proband and his mother identified the same germline c.681dup, p.(Leu228ThrfsTer33), *RUNX1* variant (Chromosome (Chr) 21, position 34834534, reference genome Hg38–Table 1 and Fig. 2). The c.681dup classification resulted as pathogenic, based on the ClinGen criteria (https://clinicalgenome.org/docs/clingen-myeloid-malignancy-expert-panel-specifications-to-the-acmg-amp-variant-interpretation-guidelines-version-2/, https://cspec.genome.network/cspec/ui/svi/doc/GN008) [11, 12]. Applied classification criteria are shown in the Table 1 of the Online Resource 1. In addition, the diagnosis of FRDA emerged in the mother and her three siblings, the patient and his brother being obligate carriers. Accordingly, the genetic test confirmed the heterozygous trinucleotide-GAA repeat expansion (1000±50) in the *FXN* gene, in the proband’s brother. Genetic counselling did not reveal any other clinical condition.

**Table 1:** Results from the NGS analysis of the proband (Hg38 reference genome)

PTS ID	Age, sex	Diagnosis	Germline variants (VAF%)	Somatic variants (VAF%)	Post-transplant	Somatic variants (VAF%)
UPN1242	43, M	AML	RUNX1 (NM_001754.5) c.681dup p.(Leu228ThrfsTer33) (46.2%) MPO (NM_000250.2) c.2031–2 A > C p.(?) (38.8%)	NF1 (NM_001042492.3) c.1019_1020insAAAATAGG p.(Val341LysfsTer2) (90%)	T-ALL	NF1 (NM_001042492.3) c.1019_1020insAAAATAGG p.(Val341LysfsTer2) (95.3%) FBXW7 (NM_001349798.2) c.1394G > A p.(Arg465His) (95.7%) NOTCH1 (NM_017617.5) c.4754T > C p.(Leu1585Pro) (45%)

Myeloid and Lymphoid NGS panels used for the somatic variants screening identified also the germline *RUNX1* variant

VAF, Variant Allele Fraction; AML, Acute Myeloid Leukemia; T-ALL, T lymphocytes-Acute Lymphoblastic Leukemia

Five months after diagnosis, in May 2023, the proband underwent BM transplant from a 9/10 Matched Unrelated Donor (MUD). However, after three months, the BM was massively infiltrated by 97% of blasts positive for immature and T cell lineage antigens (CD34, TdT, weak CD38, CD7, cyCD3, CD2, weak CD5, CD4, CD1 and CD10). Around 25% of these blasts co-expressed weak CD13, and HLA-DR antigens. This pattern was compatible with ALL of T-lineage (T-ALL). Histological examination reported a massive infiltration by hypo-a-granular blasts (90%). Immunohistochemistry showed TdT, cyCD3, CD2, CD7, CD5, cyCD79a, partial CD1a and CD4 positivity; supporting the diagnosis of T-ALL. Notably, the host origin of this leukemia was supported by the identification of the familial *RUNX1* mutation in BM cells. Salvage therapy was started according to the GIMEMA ALL 1913 protocol. At early evaluation, 20 days after chemotherapy, 98% of blasts persisted. The patient died at the end of August 2023.

## Genotypic studies

**Fig. 3:**
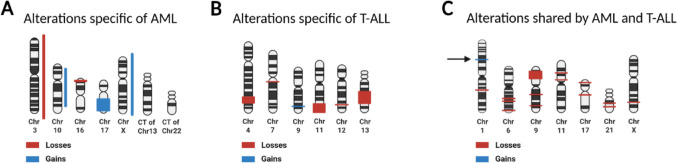
Schematic representation of the genetic characterization of the proband leukemias through SNP array. The losses are reported in red and the gains in blue. Anomalies specifically associated with AML (**A**) or T-ALL (**B**).** C** Anomalies common to both AML and T-ALL; the arrow indicates the unique germline anomaly found also in the proband mother and his healthy brother. AML: Acute Myeloid Leukemia, T-ALL: T Lymphocytes-Acute Lymphoblastic Leukemia, CT: Chromotripsis, Chr: Chromosome. Created in BioRender. Lema, A. (2025) https://BioRender.com/xfuy56w

The genomic characterization of the proband, his mother, and his healthy brother was carried out by Next Generation Sequencing (NGS) and Single Nucleotide Polymorphisms array (SNPa -Affymetrix). Results from proband and his mother are shown in detail in Table 1; Fig. 3; and in Table 1 of Online Resource 2, respectively (See material and methods in Online Resource 3). In addition to the *RUNX1* variant, the proband and his mother shared an intronic *MPO* (c.2031–2A>C, p.(?)) variant, currently classified as Variant of Uncertain Significance (VUS) by the American College of Medical Genetics and Genomics, ACMG, Varsome database (v.13.14.3, https://varsome.com) [13, 14]. The classification criteria are also reported in Table 2 of the Online Resource 1. Both *RUNX1* and *MPO* variants were absent in the healthy brother. Notably, the proband showed also a pathogenic variant at the *NF1* gene (c.1019_1020insAAAATAGG, p.(Val341LysfsTer2)) that was maintained at the time of the post-transplant T-ALL (Table 1). However, we excluded its germline origin because absent in both the mother and the healthy brother, as well as in DNA obtained from proband nails. Moreover, no signs of Neurofibromatosis, associated with *NF1* gene mutations, were observed in the patient (Online Mendelian Inheritance in Man, OMIM^®^. Johns Hopkins University, Baltimore, MD. MIM Number: #162200: 06/26/2024: World Wide Web URL: https://omim.org/). As expected in T-ALL, additional somatic variants affecting *NOTCH1* (c.4754T>C, p.(Leu1585Pro)) and *FBXW7* (c.1040G>A, p.(Arg465His)) (Table 1) were found in the proband’s post-transplant leukemia. Somatic variants with a low allelic frequency at *TET2*, *DNMT3A*, and *FAT1* genes were identified in the only available maternal sample in remission after chemotherapy (Table 1). The low allelic frequency of these genes suggested clonal myeloid hematopoiesis that is frequently associated with *RUNX1* mutations [15, 16].

Interestingly, SNPa provided further insights on both germline and somatic changes (Fig. 3 and raw data in the Online Resource 4). First, one gain at 1p31.1 was present in the proband, his mother and in the healthy brother, indicating that at least this genomic event was of germline origin and unlikely related to the leukemia phenotype (Fig. 3). Expected further somatic variants at *RUNX1*, in particular mutations or deletions involving the second allele [9, 10], were not identified in the genotypic characterization of the proband via NGS and SNPa. Additionally, SNPa clarified that homozygosity of both the *NF1* and the *FBXW7* variants (VAF ≥90%) was due to the concomitant somatic deletions at 17q11 and 4q31 (Fig. 3), respectively. Fluorescence In Situ Hybridization (FISH) confirmed these deletions and revealed also a small clone (around 8%) with a rearranged *BCL11B* gene (Materials and methods in the Online Resource 3 and Figure in the Online Resource 5).

## Discussion

In the present Family, the dominantly inherited leukemia segregated with a pathogenic germline c.681dup *RUNX1* gene variant. Notably, typical FDP features, such as thrombocytopenia, bleeding, and/or bruising, did not emerge from our patient medical history. Both the mother and the maternal grandfather were old leukemic patients (≥70 years), while the proband developed the leukemia at 43 years of age. In our proband the myeloid leukemia found at diagnosis was followed by a T-lymphoid leukemic phenotype after transplantation (Table 1). Remarkably, heterozygous germline *RUNX1* mutations are involved in predisposition to both AML and T-ALL. The presence of shared genetic markers at SNPa and of the same *NF1* gene mutation, in both AML at diagnosis and T-ALL after transplant is a noteworthy observation (Table 1; Fig. 3), suggesting a common origin from an immature leukemic cell able to activate both the myeloid or the T lymphoid differentiation program, respectively at diagnosis and post-transplantation. Interestingly, we previously observed these pathobiological features in acute leukemia with activated *BCL11B* gene [17]. Thus, our hypothesis is that the genetic markers shared by both AML and T-ALL cells identify the leukemic stem cell, while the different additional aberrations seen at diagnosis and after transplant are the biomarkers of the clonal evolution underpinning the different phenotypes.

As far as we know the c.681dup *RUNX1* gene variant has never been described. It is generated by a single duplication of a thymine responsible for frameshift and an anticipated stop-codon and it is located between the RUNT and TAD domains, a region rarely affected by pathogenic variants [6, 18]. The consequent frameshift involves all the *RUNX1* isoforms, maintaining only the RUNT domain in all three cases, with the TAD and Inhibitory domain going lost (Fig. 2). It is well established that variants introducing premature termination codons (PTCs) can trigger nonsense-mediated mRNA decay (NMD). For NMD to occur, however, the PTC must meet specific criteria, particularly it should not be located in the last exon or in a site less than 50–55 nucleotides upstream of the final exon–exon junction. Moreover it should be located > 200 bp downstream of the start codon [19–21]. Thus, in our *RUNX1* variant NMD is predicted to affect the two longest isoforms (B and C), whereas NMD is not expected in the shortest isoform (A), with the PTC lying within 50 nucleotides from the last exon–exon junction. Consequently, haploinsufficiency is likely affecting the isoform B and the isoform C which is mostly expressed in the whole blood (from the GTEx Portal on 02/17/2026.).

This rare *RUNX1* event in our family coexisted with FRDA, a rare recessive genetic condition. In particular, the mother was affected by FRDA, while both the proband and his brother were healthy carriers. This disease in almost all cases arises from a homozygous expansion of a nucleotide triplet (GAA) within the first intron of the *FXN* gene, encoding for a protein that is involved in the formation of iron-sulphur proteins at mitochondrial level [22]. Its reduced expression has been correlated to telomere dysfunction in FRDA cells. Moreover, alterations at DNA repair pathways have also been described in FRDA [23]. As far as we know, however, the leukemia risk, if any, in Families with *FXN* gene mutations is unknown. Indeed, in our Family, the leukemia is attributable to the *RUNX1* germline variant rather than to the *FXN* repeat expansion.

Lastly, a third germline variant, i.e., the intronic c.2031–2A>C *MPO* variant, was found only in patients bearing the *RUNX1* variant and the leukemia phenotype. *MPO* germline variants were suggested to behave as predisposing factors to myeloid neoplasias [24], and decreased *MPO* transcription in cells bearing the c.2031–2A>C has been observed in the hematological malignancies [25]. However, the classification of the c.2031–2A>C is still challenging. It is “conflicting” from ClinVar [26], while, by adopting ACMG criteria [14], the Varsome database (v.13.14.3, https://varsome.com) [13], defines the variant of uncertain significance (Table 2 of the Online Resource 1). Its role in leukemia predisposition, if any, remains to be clarified.

In conclusion, we have described a unique Family with autosomal dominant leukemia segregating with a new rare germline pathogenic *RUNX1* variant (c.681dup, p.(Leu228ThrfsTer33)). Genomic investigations provided us with in depth characterization of the AML and the T-ALL phenotypes characterizing the proband leukemias respectively at diagnosis and after transplantation.

## Supplementary Information

Below is the link to the electronic supplementary material.

Supplementary Material 1

Supplementary Material 2

Supplementary Material 3

Supplementary Material 4

Supplementary Material 5

## Data Availability

All data generated in this study are publicly available in the Gene Expression Omnibus (GEO) database (http://www.ncbi.nlm.nih.gov/geo/) with the accession number GSE305159.
