# Supplementary material for: A new c.681dup RUNX1 variant in familial leukemia
Source: Fam Cancer. 2026 Apr 6;25(2):37. doi: 10.1007/s10689-026-00550-7 (PMC13053502; doi:10.1007/s10689-026-00550-7)
Supplement: Supplementary file 1 — Supplementary Material 1 [file 10689_2026_550_MOESM1_ESM.pdf]

A New c.681dup *RUNX1* Variant in Familial Leukemia

JOURNAL: **FAMILIAL CANCER**

AUTHORS:

Maria Crocioni[1], Carlotta Nardelli[1], Anair G. Lema Fernandez[1], Valentina Bardelli[1], Valentina Pierini[1], Caterina Matteucci[1], Eloise Beggiato[2], Matteo Olivi[3], Valentina Vigliani[4], Alessandra Pelle[5], Giuseppe Lanzarone[2], Cristina Mecucci[1]

CORRESPONDING AUTHOR DETAILS

AFFILIATION:

Prof. Cristina Mecucci MD PhD Centro di ricerca Emato-Oncologiche University of Perugia piazzale Menghini 9, 06132 Perugia, Italy.

EMAIL:

cristina.mecucci@unipg.it

Classification of the *RUNX1* (c.681dup, p.L228Tfs*33) variant according to ClinGen MM-VCEP criteria. Columns "Interpretation" and "Comments" are described according to the familial features

ACMG/AMP criteria code	Original ACMG/AMP rule summary	Specification	Interpretation	Comments	Score*
**PVS1**	Null variant in gene where LOF is a known mechanism of disease	Gene-specific strength	*Very strong* – per modified *RUNX1* PVS1 decision tree for SNVs, indels and CNVs and table of splicing effect	*RUNX1* LOF variants are common mechanism of disease in FPD/AML. Three major isoforms (A, B and C) are expressed by use of two promoters in alternative splicing. NMD affects isoforms B and C. The shortest isoform A is not predicted to undergo NMD, therefore, the interpretation is downgraded to PVS1_Strong (score: +4)**.	+8
**PS4**	The prevalence of the variants in affected individuals is significantly increased compared with the prevalence in control subjects	Disease-specific strength	*Supporting* - One proband meeting *RUNX1*-phenotypic criteria	The affected individual fits one of the *RUNX1*-phenotypic criteria (diagnosis of hematological malignancies) and the variant is absent from gomAD (overall population)	+1
**PM2**	Absent from control subjects	General recommendation	*Supporting* – per original ACMG/AMP guidelines	Variant is completely absent from all population databases***.<br>The mean coverage of *RUNX1* in the population database used should be at least 20X	+1

Table 1. *Table reconstructed on our Family features following the MM-VCEP ACMG/AMP specifications for RUNX1 variants* [1, 2]. **The scores were calculated following the guidelines reported by Tavtigian et al. (2020)* [3]. *** Due to the PVS1_Strong interpretation, the variant classification only in the case of the shortest isoform (A) results as "Likely Pathogenic" (total score: +6). ***The population databases examined include 1000 Genomes, GnomAD (including ExAC), RGC Million Exome Variant Browser, TOPMed, dbSNP and dbVAR. ACMG: American College of Medical Genetics and Genomics, AMP: Association for Molecular Pathology, PVS: Pathogenic Very Strong, PS: Pathogenic Strong, PM: Pathogenic moderate, LOF: Loss of Function, PTS: points.*

Classification of the *MPO* (c.2031-2A>C , p.?) variant according to ACMG criteria from Varsome database (v.13.14.3, https://varsome.com) slightly modified.

ACMG criteria code	Original ACMG rule summary	Interpretation	Score
**PVS1**	Null variant (nonsense, frameshift, canonical ±1 or 2 splice sites, initiation codon, single or multiexon deletion) in a gene where LOF is a known mechanism of disease.	*Strong*	+4
**PS3**	Well-established in vitro or in vivo functional studies supportive of a damaging effect on the gene or gene product.	*Moderate*	+2
**PP5**	Reputable source recently reports variant as pathogenic, but the evidence is not available to the laboratory to perform an independent evaluation	*Moderate*	+2
**BS2**	Observed in a healthy adult individual for a recessive (homozygous), dominant (heterozygous), or X-linked (hemizygous) disorder, with full penetrance expected at an early age.	*Strong*	-4

Table 2. *Table reporting the ACMG criteria of MPO variant (c.2031-2A>C , p.?) classification in the Varsome database (v.13.14.3, https://varsome.com)* [4]. *The scores were calculated by Varsome following the guidelines of Tavtigian et al. (2020)* [3]. *ACMG: American College of Medical Genetics and Genomics, PVS: Pathogenic Very Strong, PS: Pathogenic Strong, PP: Pathogenic supporting, BS: Benign Strong LOF: Loss of Function.*

Reference

1. Luo X, Feurstein S, Mohan S, et al (2019) ClinGen Myeloid Malignancy Variant Curation Expert Panel recommendations for germline RUNX1 variants. Blood Advances 3:2962–2979. https://doi.org/10.1182/bloodadvances.2019000644

2. Feurstein S, Adegunsoye A, Mojsilovic D, et al (2020) Telomere biology disorder prevalence and phenotypes in adults with familial hematologic and/or pulmonary presentations. Blood Advances 4:4873–4886. https://doi.org/10.1182/bloodadvances.2020001721

3. Tavtigian SV, Harrison SM, Boucher KM, Biesecker LG (2020) Fitting a naturally scaled point system to the ACMG/AMP variant classification guidelines. Human Mutation 41:1734–1737. https://doi.org/10.1002/humu.24088

4. Kopanos C, Tsiolkas V, Kouris A, et al (2019) VarSome: the human genomic variant search engine. Bioinformatics 35:1978–1980. https://doi.org/10.1093/bioinformatics/bty897
