# Supplementary material for: A new c.681dup RUNX1 variant in familial leukemia
Source: Fam Cancer. 2026 Apr 6;25(2):37. doi: 10.1007/s10689-026-00550-7 (PMC13053502; doi:10.1007/s10689-026-00550-7)
Supplement: Supplementary file 2 — Supplementary Material 2 [file 10689_2026_550_MOESM2_ESM.pdf]

A New c.681dup *RUNX1* Variant in Familial Leukemia

JOURNAL: **FAMILIAL CANCER**

AUTHORS:

Maria Crocioni[1], Carlotta Nardelli[1], Anair G. Lema Fernandez[1], Valentina Bardelli[1], Valentina Pierini[1], Caterina Matteucci[1], Eloise Beggiato[2], Matteo Olivi[3], Valentina Vigliani[4], Alessandra Pelle[5], Giuseppe Lanzarone[2], Cristina Mecucci[1]

CORRESPONDING AUTHOR DETAILS

AFFILIATION:

Prof. Cristina Mecucci MD PhD Centro di ricerca Emato-Oncologiche University of Perugia piazzale Menghini 9, 06132 Perugia, Italy.

EMAIL:

cristina.mecucci@unipg.it

Table 1. Results from the NGS analysis of the proband's mother (Hg38 reference genome)

PTS ID	AGE, SEX	DIAGNOSIS	GERMLINE VARIANTS (VAF%)	SOMATIC VARIANTS (VAF%)
**UPN1342**	72, F	**AML**	***RUNX1*** (NM_001754.5) c.681dup<br>p.(Leu228ThrfsTer33) (40%)<br>***MPO*** (NM_000250.2) c.2031-2A>C<br>p.(?) (38.8%)	***TET2*** (NM_001127208.3) c.4889C>A<br>p.(Ser1630Ter) (8%)<br>***TET2*** (NM_001127208.3)<br>c.238C>T<br>p.(Gln80Ter) (1.8%)<br>***TET2*** (NM_001127208.3) c.4624C>T<br>p.(Gln1542Ter) (5.2%)<br>***DNMT3A*** (NM_022552.5) c.2711C>A<br>p.(Pro904Gln) (2.2%)<br>***FAT1*** (NM_005245.4)<br>c.6463G>T<br>p.(Val2155Phe) (7%)

Myeloid NGS panel used for the somatic variants screening identified also the germline *RUNX1* variant.
The analyses were carried out in PB at post-chemotherapy remission.
VAF: Variant Allele Fraction; AML: Acute Myeloid Leukemia; PB: Peripheral Blood
