# Supplementary material for: A new c.681dup RUNX1 variant in familial leukemia
Source: Fam Cancer. 2026 Apr 6;25(2):37. doi: 10.1007/s10689-026-00550-7 (PMC13053502; doi:10.1007/s10689-026-00550-7)
Supplement: Supplementary file 3 — Supplementary Material 3 [file 10689_2026_550_MOESM3_ESM.pdf]

A New c.681dup *RUNX1* Variant in Familial Leukemia

JOURNAL: **FAMILIAL CANCER**

AUTHORS:

Maria Crocioni[1], Carlotta Nardelli[1], Anair G. Lema Fernandez[1], Valentina Bardelli[1], Valentina Pierini[1], Caterina Matteucci[1], Eloise Beggiato[2], Matteo Olivi[3], Valentina Vigliani[4], Alessandra Pelle[5], Giuseppe Lanzarone[2], Cristina Mecucci[1]

CORRESPONDING AUTHOR DETAILS

AFFILIATION:

Prof. Cristina Mecucci MD PhD Centro di ricerca Emato-Oncologiche University of Perugia piazzale Menghini 9, 06132 Perugia, Italy.

EMAIL:

cristina.mecucci@unipg.it

Genomic Characterization

Next Generation Sequencing

Targeted NGS was performed using custom and commercial SOPHiA Genetics (Sophia Genetics, Saint Sulpice, Switzerland) panels.

Commercial Myeloid Solution panel (MYS) investigates the 30 clinically most relevant genes associated with Myelodysplastic Syndrome (MDS), Myeloproliferative Neoplasms (MPN) and Acute Myeloid Leukemia (AML) and covering complete coding sequence with ± 25 bp of exon-flanking regions. Selected genes, with exons reported between brackets, are: *ABL1* (4-9), *ASXL1* (9,11,12,14), *BRAF* (15), *CALR* (9), *CBL* (8,9), *CEBPA* (all), *CSF3R* (all), *DNMT3A* (all), *ETV6* (all), *EZH2* (all), *FLT3* (13-15,20), *HRAS* (2,3), *IDH1* (4), *IDH2* (4), *JAK2* (all), *KIT* (2,8,11,13,17,18), *KRAS* (2,3), *MPL* (10), *NPM1* (10,11), *NRAS* (2,3), *PTPN11* (3,7-13), *RUNX1* (all), *SETBP1* (4), *SF3B1* (10-16), *SRSF2* (1), *TET2* (all), *TP53* (2 11), *U2AF1* (2,6), *WT1* (6-10) and *ZRSR2* (all).

Custom lymphoid panel (CHEMA_B_V1), includes genes associated with Acute B and T cells Lymphoblastic Leukemia. The selected genes are: *AKT1, ATM, BCL11B, BRAF, CCND3, CNOT3, CREBBP, CTCF, DNM2, EED, EP300, ETV6, EZH2, FAT1, FAT3, FBXW7, FLT3, GATA3, GLI1, GLI2, GLI3, IKZF1, IL2RB, IL7R, JAK1, JAK3, KDM6A, KMT2D, KRAS, LEF1, LMO1*, (non-coding region), *LMO2* (non-coding region), *MED12, MYB, NF1, NOTCH1, NRAS, NT5C2, PHF6, PIK3CD, PIK3R1, PTCH1, PTEN, RELN, RPL10, RPL22, RPL5, RUNX1, SETD2, SH2B3, SMARCA4, SMO, STAT5B, SUZ12, TAL1* (non-coding region), *TP53, TYK2, USP7, USP9X*, and *WT1*.

The Custom Hereditary Haematological Disorders (CHHD_A_v2) consists of 63 genes, 40 related to telomere biology and 23 to known leukemia predisposing genes. The panel includes the whole coding sequence of each transcripts ± 5 bp of exon-flanking regions of the following genes: *ANKRD26*, *ASF1A*, *ASF1B*, *ATRX*, *BLM*, *CBL*, *CEBPA*, *COX4I1*, *CTC1*, *CTCF*, *DAXX*, *DCLRE1B*, *DDX41*, *DICER1*, *DKC1*, *DNAJC21*, *ETV6*, *EZH2*, *FUS*, *GAR1*, *GATA2*, *GNAS*, *LIG4*, *MBD4*, *MECOM*, *MRE11*, *NAF1*, *NBN*, *NHP2*, *NOP10*, *NSD1*, *NSD3*, *PABPN1*, *PARN*, *PIF1*, *PINX1*, *POT1*, *RAD50*, *RECQL4*, *RIF1*, *RTEL1*, *RUNX1*, *SAMD9*, *SAMD9L*, *SRP72*, *STN1*, *TEN1*, *TERC*, *TERF1*, *TERF2*, *TERF2IP*, *TERT*, *TINF2*, *USB1*, *WRAP53*, *ZEB2*. The 5'UTR region, the whole coding sequence, and ± 25 bp of exon-flanking regions of *ACD*, *ERCC6L2*, *MDM4*, *NPM1* and *ZCCHC8*. Partial coding sequence, with ± 5bp flanking region, was included in the panel for *HLTF* (exon7) and *MPO* (exons 10 and 12) genes.

For germline genomic DNA (gDNA), CD3+ lymphocytes and/or nails, when available, were used. Libraries were prepared using 200ng of gDNA following manufacturer's instructions. Once the

library pool was obtained, it was sequenced on Illumina MiSeq Sequencer (Illumina, San Diego, CA, USA) with MiSeq Reagent Kit. The obtained FASTQ files were analyzed with SOPHiA DDM software (version 5.10.54.3), reference genome GRCh37/hg19. The reference genome for the NGS results have been then updated and converted to the Hg38. Minor Allele Frequency (MAF) <0.01 and Variant Allele Fraction (VAF) ≥2% were established to filter the variants, taking into consideration only exonic, splice site and non-coding targets. The prediction of each variant was then established using the Varsome program (Saphetor, SA, Lausanne, Switzerland) referring to the guidelines of the American College of Medical Genetics and Genomics (ACMG) [1], ClinVar [2] and Association for Molecular Pathology (AMP) databases [1].

Single Nucleotide Polymorphisms Array (SNPa)

SNPa inform copy number variations, by using probes specific for the SNPs distributed along the whole genome, detecting Copy Number Variations (CNV), Loss of Heterozygosity (LOH), and Copy Neutral LOH.

DNA samples from AML diagnosis and post-transplant T-ALL diagnosis of the proband were analysed with Cytoscan HD Array (Affymetrix/Thermofisher, Santa Clara, CA, USA) following the manufacturer's protocol. Chromosome Analysis Suite (ChAS) software was used for the data elaboration with GRCh37/hg19 genome as reference and filter settings at 200k, 50 markers for CNVs and 10Mb, 50 markers for cnLOH. Database of Genomic Variants [3] was used to exclude polymorphic copy number variations.

Interphase Fluorescent In Situ Hybridization (FISH)

Interphase FISH was conducted on the proband BM sample at T-ALL relapse to confirm the deletion of C*DKN2AB* (Vysis CDKN2AB Spectrum Orange (SO)/CEP9 Spectrum Green (SG), Abbott Molecular, Rome, Italy) and *JAK2* (RP11-39K24 SO/RP11-125K10 SG) at 9p; *RB1* (RP11-305D15 SG/ RP11-174I10 SO) and *D13S319* (Vysis LSI D13S319 SO, 13q34 Spectrum Aqua (SA), CEP12 SG; CLL FISH probe kit , Abbot Molecular) at 13q; *WT1* (RP1-74G1) at 11p13; 11q22-*ATM* (Vysis LSI ATM SG, LSI TP53 SO; CLL FISH probe kit, Abbot Molecular) and *KMT2A* (Vysis LSI MLL Dual Collor, Break Apart, Abbot Molecular) at 11q; *TP53* (Vysis LSI MLL Dual Collor, Break Apart, Abbot Molecular) at 17p*;* and *NF1* (RP5-926B9 SO(4)/RP5-1002G3 SG [4]) and *SUZ12* (RP11-279B10 SO(4)/RP11-640N20 SG [4]) at 17q (data not shown). Moreover, also the *BCL11B* (RP11-74H1 SG/RP11-431B1 SO) rearrangement with an unknown partner was identified by interphase FISH (**Supplementary Figure 1**).

FISH probes evaluating *JAK2*, *RB1, WT1,* and *BCL11B* were directly labelled and were designed from BACs (RPCI-11 Human Male BAC Library and Caltech BAC CTB, CTC, and CTD Libraries), P1-derived artificial chromosomes (PACs; RPCI-1 and RPCI-5; Human Male PAC Library), and Fosmids (WIBR-2 Human Fosmid Library; National Center for Biotechnology Information, https://www.ncbi.nlm.nih.gov/genome/gdv; UCSC Genome Browser, University of California, Santa Cruz, https://genome.ucsc.edu, last accessed July 30, 2019).

SUPPLEMENTARY REFERENCES

1. Richards S, Aziz N, Bale S, Bick D, Das S, Gastier-Foster J, Grody WW, Hegde M, Lyon E, Spector E, Voelkerding K, Rehm HL (2015) Standards and guidelines for the interpretation of sequence variants: a joint consensus recommendation of the American College of Medical Genetics and Genomics and the Association for Molecular Pathology. Genetics in Medicine 17:405–424. https://doi.org/10.1038/gim.2015.30

2. Landrum MJ, Lee JM, Riley GR, Jang W, Rubinstein WS, Church DM, Maglott DR (2014) ClinVar: public archive of relationships among sequence variation and human phenotype. Nucleic Acids Res 42:D980–D985. https://doi.org/10.1093/nar/gkt1113

3. MacDonald JR, Ziman R, Yuen RKC, Feuk L, Scherer SW (2014) The Database of Genomic Variants: a curated collection of structural variation in the human genome. Nucl Acids Res 42:D986–D992. https://doi.org/10.1093/nar/gkt958

4. La Starza R, Pierini V, Pierini T, Nofrini V, Matteucci C, Arniani S, Moretti M, Lema Fernandez AG, Pellanera F, Di Giacomo D, Storlazzi TC, Vitale A, Gorello P, Sammarelli G, Roti G, Basso G, Chiaretti S, Foà R, Schwab C, Harrison CJ, Van Vlierberghe P, Mecucci C (2020) Design of a Comprehensive Fluorescence in Situ Hybridization Assay for Genetic Classification of T-Cell Acute Lymphoblastic Leukemia. The Journal of Molecular Diagnostics 22:629–639. https://doi.org/10.1016/j.jmoldx.2020.02.004
