# Supplementary material for: A new c.681dup RUNX1 variant in familial leukemia
Source: Fam Cancer. 2026 Apr 6;25(2):37. doi: 10.1007/s10689-026-00550-7 (PMC13053502; doi:10.1007/s10689-026-00550-7)
Supplement: Supplementary file 5 — Supplementary Material 5 [file 10689_2026_550_MOESM5_ESM.pdf]

A New c.681dup *RUNX1* Variant in Familial Leukemia

JOURNAL: **FAMILIAL CANCER**

AUTHORS:

Maria Crocioni[1], Carlotta Nardelli[1], Anair G. Lema Fernandez[1], Valentina Bardelli[1], Valentina Pierini[1], Caterina Matteucci[1], Eloise Beggiato[2], Matteo Olivi[3], Valentina Vigliani[4], Alessandra Pelle[5], Giuseppe Lanzarone[2], Cristina Mecucci[1]

CORRESPONDING AUTHOR DETAILS

AFFILIATION:

Prof. Cristina Mecucci MD PhD Centro di ricerca Emato-Oncologiche University of Perugia piazzale Menghini 9, 06132 Perugia, Italy.

EMAIL:

cristina.mecucci@unipg.it

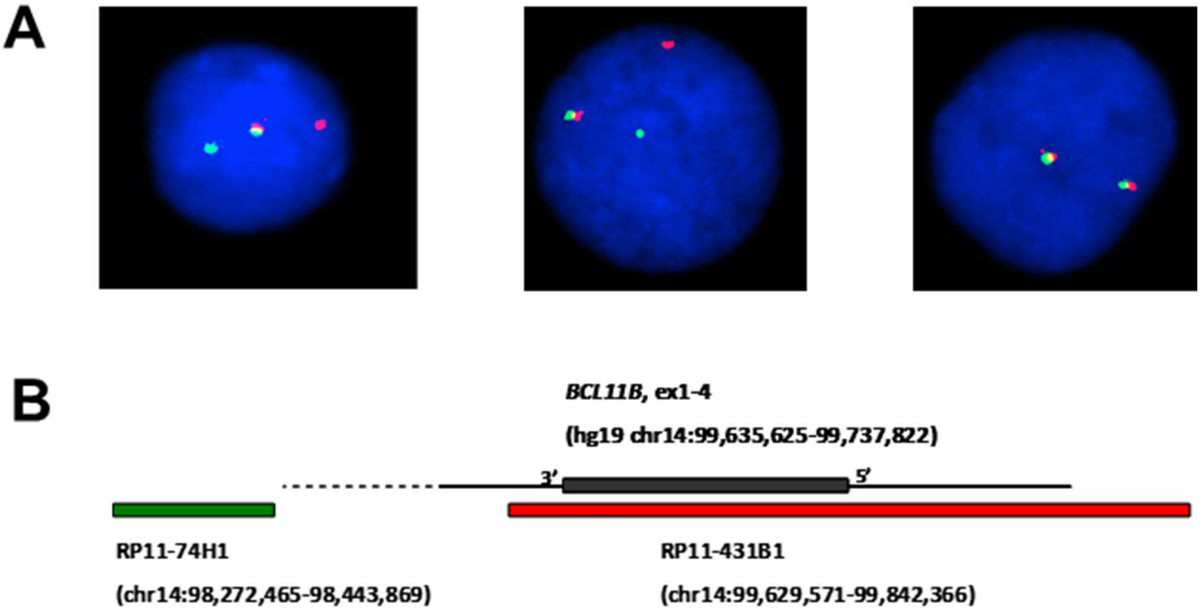

Figure 1: ***A.*** *Nuclei hybridized with BCL11B/14q32 break-apart FISH probe (RP11-74H1 SpectrumGreen/RP11-431B1 SpectrumOrange): from left to right first and second images show abnormal hybridization pattern consisting of one fusion signal and separate red and green signals, compatible with BCL11B locus rearrangement. Right image shows a normal nucleus with two fusion signals.* ***B.*** *Schematic representation of BCL11B/14q32 break-apart FISH probe: RP11-74H1 (green) maps about 1,2 Mb centromeric to 3' BCL11B. RP11-431B1 (orange) spans the entire gene and extends telomerically. Figure not to scale.*
